# Section level search functionality in Europe PMC

**DOI:** 10.1186/s13326-015-0003-7

**Published:** 2015-03-10

**Authors:** Şenay Kafkas, Xingjun Pi, Nikos Marinos, Francesco Talo’, Andrew Morrison, Johanna R McEntyre

**Affiliations:** European Molecular Biology Laboratory, European Bioinformatics Institute (EMBL-EBI), Wellcome Trust Genome Campus, Hinxton, Cambridge, CB10 1SD, United Kingdom

**Keywords:** Text mining, Section, Information retrieval

## Abstract

**Background:**

As the availability of open access full text research articles increases, so does the need for sophisticated search services that make the most of this new content. Here, we present a new feature available in Europe PMC that allows selected sections of full text articles to be searched, including figures and reference lists. Users can now search particular parts of an article, reducing noise and allowing fine-tuning of searches.

**Results:**

To the best of our knowledge, Europe PMC is the first service that provides a granular literature search by allowing users to target their search to particular sections of articles. This new functionality is based on a heuristic algorithm that identifies and categorises article sections into 17 pre-defined categories based on the section heading. The tagger’s performance is measured against a manually curated dataset consisting of 100 full text articles with an F-score of 98.02%.

**Conclusions:**

The section search is available from the advanced search within Europe PMC (http://europepmc.org). The source code is freely available from http://europepmc.org/ftp/oa/SectionTagger/.

**Electronic supplementary material:**

The online version of this article (doi:10.1186/s13326-015-0003-7) contains supplementary material, which is available to authorized users.

## Background

Life science research articles are narrative accounts of research findings, usually describing methods, experimental results, and providing scientific context to the new work reported. Most typical research articles are structured into sections (segments), most often represented by a logical sequence, known as IMRAD - “Introduction”, “Materials & Methods”, “Results” and “Discussion” [1]. However, synonyms of these typical section titles are frequently used in articles, according to different journal styles. Furthermore, other types of sections are common, such as “Case Report” in clinical journals, or additional sections such as “Funding Sources”. These sections provide useful context for the human reader’s understanding of the findings described.

The availability of full text articles online provides the opportunity to develop deep search over the complete article, not just the abstract. While this extends the content available for searching, it can also unfortunately add significant noise in the results returned. For example, for searches that order results by publication date or citation count, the results at the top of the list can have little bearing on the original search term if that term is found only in the Reference list.

There are a few free-to-use services that provide biomedical literature search services on full-text documents, for example, PubMed Central (http://www.ncbi.nlm.nih.gov/pmc), Google Scholar (http://scholar.google.co.uk/), BioText Search Engine (http://biosearch.berkeley.edu) and Yale Image Finder (YIF) (http://krauthammerlab.med.yale.edu/imagefinder/). However, to the best of our knowledge, neither PubMed Central nor Google Scholar allows users to limit searches to, or exclude, particular sections of articles. BioText allows users to limit searches to figure captions and tables, and YIF only allows users to limit searches to figure captions. At Europe PMC (http://europepmc.org) [2], we have implemented a comprehensive section-level search feature that is applied to incoming full text articles daily, and have exposed it to users both within the default search on the Europe PMC website, and within the Advanced Search form.

## Implementation

### Implementation details

This section-level search feature has been implemented as a component of the existing Europe PMC full text infrastructure. As the database is updated with new full text content, a rule-based section tagger, developed to identify the sections of full text articles, is deployed prior to Lucene indexing (http://lucene.apache.org/). Further implementation details of the section tagger are provided below.

### Section categorisation

In total 17 section category types have been identified as frequently occurring, based on an analysis of content of structured section headers (section headers are tagged by using the <title> XML element, e.g. <title> Methods</title>) appearing in the XML of the open access (OA) set of Europe PMC articles. The pre-selected categories are: Introduction & Background, Materials & Methods, Discussion, Conclusion & Future Work, Case Study, Acknowledgement & Funding, Author Contribution, Competing Interest, Supplementary Data, Abbreviations, Key words, References, Appendix, Figures, Tables, and Other where the section “Other” is used for sections that cannot be categorised into one of other 16 categories and including abstracts. This allows all articles that can be parsed to be included (i.e. all XML documents).

The categorisation rules are based on the manual analysis of a section header terminology created from the top 150 most frequently occurring section headings appearing in the OA-PMC set. The distribution of the natural language section headings complies with Zipf's law [3] (Additional file 1 Figure S1, Additional file 2: Table S2), that is, the top 150 most frequently occurring headings make up the majority (85.48%) of all the heading variations found in the OA-PMC set. A list of the rules used is provided as supplementary information (see Additional file 1: Table S1), but a typical example is “annotate the identified section as *Conclusion & Future Work*” if the section heading matches with: (conclusion | key message | future | summary | recommendation | implications for clinical practice | concluding remark)”. Section headings that fall into more than one category (e.g. “Results and Discussion”) are assigned to all matched categories.

### The interface

**Figure 1:**
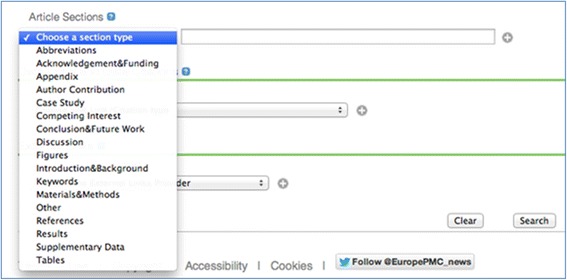
**The interface for section searching in the Europe PMC Advanced Search page (** http://europepmc.org/advancesearch **).**

The section-level search feature is provided in two ways: (1) in the default full-text search on the Europe PMC website, in which we now exclude articles from search results that contain the search terms *only* in the “References” section; (2) From the Advanced Search (http://europepmc.org/advancesearch). In the advanced search interface, a choice of 17 different section types is provided in a drop-down menu (see Figure 1), which can be combined multiply, as well as with other elements on the form through the use of typical Boolean logical terms (AND, OR, NOT). The default search behaviour to ignore hits to reference lists only can also be over-ridden here by selecting the References section. “Abstract” is not listed as a separate section category in this menu, since abstract searching is already possible via the default main search, which covers all 24 million PubMed records as well as the 3 million full text articles in Europe PMC. Further information on how to search Europe PMC is provided in Europe PMC Help (http://europepmc.org/Help).

## Results and discussion

### Analysis of the open access full text articles

**Figure 2:**
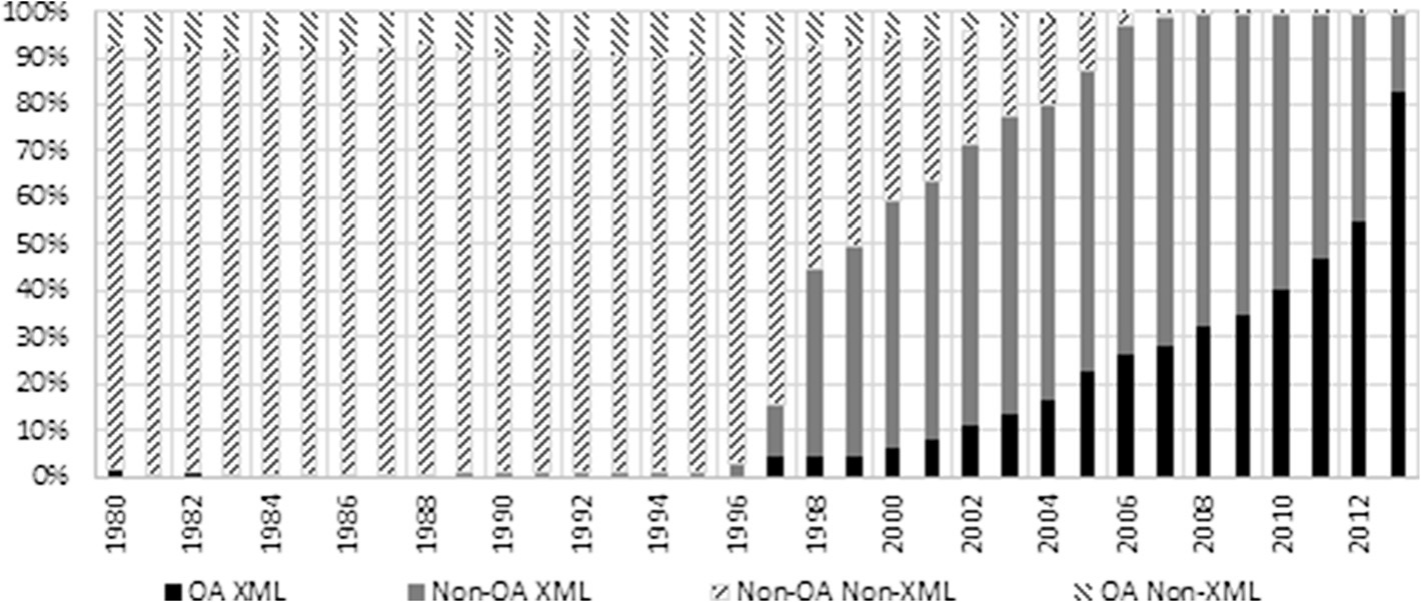
**Distribution of XML to non-XML documents, including OA status, by publication year.** This figure shows the distribution of XML to non-XML documents available in Europe PMC including OA status by publication year. The section tagger operates on the full text articles provided in XML format only. The figure shows that XML-formatted documents make up close to 100% of content available in Europe PMC that has been published in the last 7 years, which means that only a small minority of recent articles available in Europe PMC are missed.

The section tagger only operates on the full text articles that are available as XML, since OCR (scanned) content lacks parsable section headings. However, Figure 2 shows that XML-formatted documents make up close to 100% of Europe PMC content published in the last 7 years.

**Figure 3:**
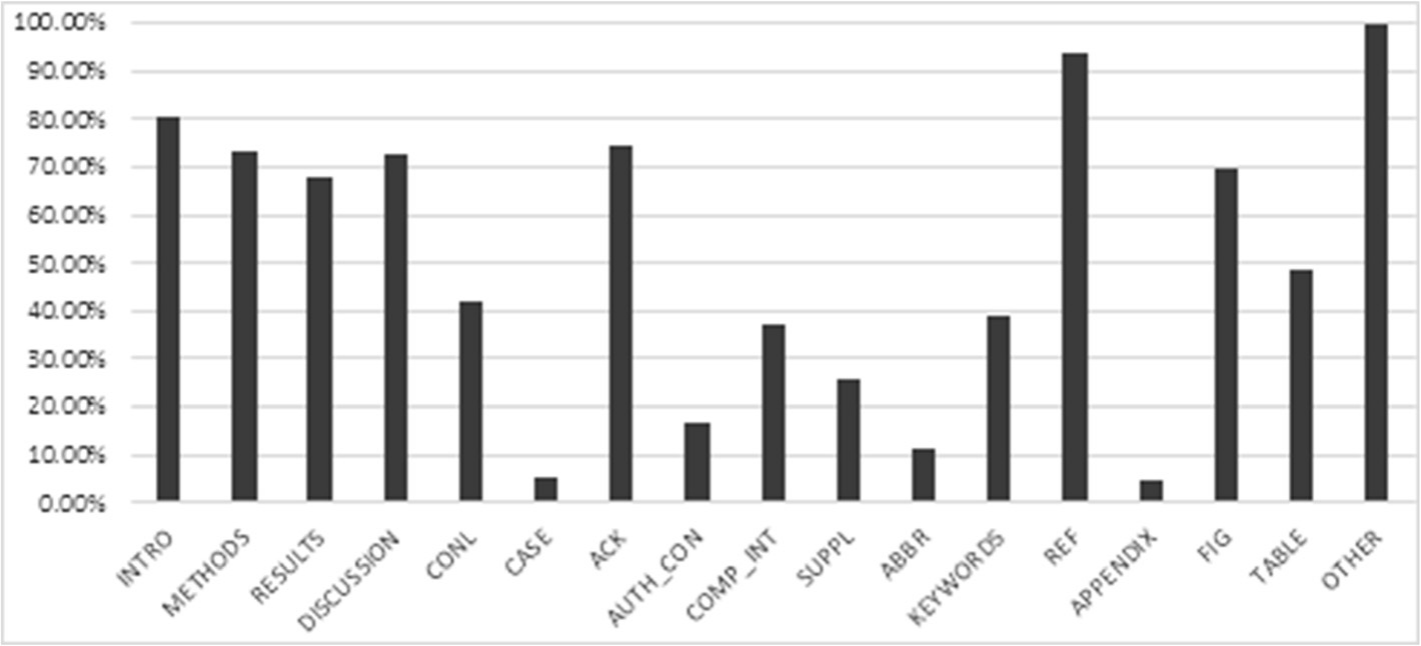
**Distribution of sections in OA articles.** This figure shows the distribution of 17 different pre-selected sections present in OA articles (INTRO: Introduction & Background, CONCL: Conclusion & Future Work, CASE: Case Report, SUPPL: Supplementary Data, KEYWORDS: Keyword, ABBR: Abbreviation, METHODS: Materials & Methods, AUTH_CON: Author Contribution, COMP_INT: Competing Interest, ACK: Acknowledgement & Funding, REF: References, FIG: Figures, TABLE: Tables, APPENDIX: Appendix, RESULTS: Results, DISCUSSION: Discussion, OTHER: Other). The results show that at least one of the typical IMRAD section types (Introduction, Materials and Methods, Results and Discussion) are found in 68-80% of articles.

We analysed the coverage of the section tagger on the OA article set (http://europepmc.org/ftp/archive/v.2013.12/oa/) (Figure 3). The results show that at least one of the typical IMRAD section types are found in 68-80% of articles.

### Performance evaluation

The tagger’s performance was estimated manually on a randomly selected set of 100 full-text articles with a Precision of 99.84%, a Recall of 96.27% and an F-score of 98.02%. The distribution of the section title frequencies in the 100 article set also complies with the Zipf's law (Additional file 1: Figure S2, Additional file 2: Table S2), which shows that it is a good representation of the whole OA-PMC set. The set of 100 full text articles was manually annotated by a single curator (ŞK). The tagger achieves a high precision but probably at the expense of recall due to missing section annotations (false negatives). This is typically because the section heading in the article is unusually worded, and therefore does not match any rule for inclusion in one of the 17 categories. For example, the section titled: “Source data and the content of the database” (PMC1347389) could be categorised as “Materials & Methods”, however, it is missed by our tagger and is therefore categorised as “Other”. These ‘custom’ titles are difficult to identify automatically.

It is not possible to directly compare our tagger with most of the existing studies concerning section identification, since they have focused on automatic classification of sentences into pre-defined sections, with the aim of aiding other text-mining tasks such as information extraction and text summarisation [4-6]. Some other studies have focused on categorising section headings of electronic health records [7,8]. However, our approach is based on the categorisation of the biomedical research article sections given their headers at the discourse level (not at the sentence level). On the other hand, there are a few systems that provide section level search functionality. The BioText search engine [9] identifies figure captions and tables in full text and allows users to limit searches by these two fields while YIF allows users to limit searches by figure captions only [10]. Like our service, BioText and YIF similarly operate on OA XML documents, and BioText identifies figure captions and tables by using XML parsing methods. However, YIF uses image-processing techniques to identify figure captions. By contrast, our service identifies 17 different section categories providing comprehensive coverage of the full article, including figures and tables. The entire document is returned in the Europe PMC service, as opposed to the stand-alone figures returned by BioText and YIF.

### Use cases

The following use cases of this new feature are already known to us, and we expect that more will emerge as the tool becomes more widely known:

1. It is used in the Europe PMC search engine to filter out hits to the References sections only. The default Europe PMC search filters out records when the search term appears only in the References section. Should the user want to search the References section, they can explicitly indicate this requirement in their query via the Advanced Search form. (For example, the query “mTOR OR REF:mTOR” will retrieve articles that contain the term “mTOR”, including those records in which mTOR is found only in the References section).
2. Searching specific article sections to find the most relevant articles for a given term type. An example is to ensure that a reagent name (e.g. protein or cell line) is mentions in the Methods section. To illustrate this point, a search for the protein mTOR, ("mTOR") AND PUB_YEAR:2012, returned 4,371 full text articles published in 2012 from Europe PMC. However, only 645 full text articles were returned when mTOR was specified to occur in the Methods sections (METHODS:"mTOR") AND PUB_YEAR:2012 (search date: 01/02/2015). Articles that mentioned mTOR in other sections but not in the Methods section where therefore removed from the search. This is a useful way of filtering out articles that potentially only mention a term in an introductory or comparative statement.
3. Focussing searches on figure legends to find graphics relevant to a search term. In some cases, specifying searches in figure captions could help to reduce the number of retrieved articles significantly. For example, a search for "protein structure" returned 37,149 full text articles. However, specifying that the term should be found in figure captions, FIG:"protein structure" returned 3,678 articles only (search date: 4/08/2014).
4. Incorporation of the section tagger into ContentMine (http://contentmine.org): The ContentMine project aims to develop tools that allow extraction of facts from scientific articles and figures. The section tagger is being integrated into this project to add structure to documents before text mining is applied.

## Conclusions

Here, we presented a new search feature of Europe PMC that enables users to search articles by section type. This is based on a new rule-based section-tagging step prior to Lucene indexing. The section tagger identifies and categorises article section headers into pre-selected section types. The aim of this functionality is to help users fine-tune full-text searches more usefully.

In the future, we plan to improve the system (perhaps by exploring machine learning approaches) so that sections that currently do not get categorised can be assigned more frequently. This would improve the tagger’s recall performance and allow it to be applied to a wider set of articles. Furthermore, we would also like to explore the further development of the Europe PMC interface to make the use of section-limited searching more discoverable to the Europe PMC users, for example, by providing filters for figure legend searching in the context of the main search, or returning text only from the section targeted, rather than the complete article.

### Availability and requirements

The section search feature is available from the Advanced Search within Europe PMC (http://europepmc.org). Please see our help page for the usage and search syntax description (http://europepmc.org/Help#searchselsections).

The Section Tagger’s source code is freely available from http://europepmc.org/ftp/oa/SectionTagger/. The code is written in the Perl scripting language (Perl 5 or above is required).

## Additional files

Additional file 1:
**This MS Word file contains a table that presents the rules used for section categorisation and two figures that represent the distribution of section titles in the full OA set, as well as in the 100-article manually-checked set, demonstrating that both sets comply with Zipf's law, and demonstrating that the 100-article test set is representative of the complete set. Table S1.** Rules Used for Section Categorisation. **Figure S1.** Section Title Frequencies in the Open Access set. **Figure S2.** Section Article Frequencies in the 100 Article Test Set. Additional file 2:
**This MS excel file contains the data used to generate Figures S1 and S2 provided from Additional file** 1 **.** This data covers the frequencies of sections in the OA set as well as the 100-article test set. **Table S2.** Frequency and Section Title.

## Abbreviations

IMRAD: “Introduction”, “Materials & Methods”, “Results” and “Discussion”
OA: Open Access
XML: eXtensible Markup Language
