# Supplementary material for: Section level search functionality in Europe PMC
Source: J Biomed Semantics. 2015 Mar 10;6:7. doi: 10.1186/s13326-015-0003-7 (PMC4359544; doi:10.1186/s13326-015-0003-7)
Supplement: Additional file 1: — This MS Word file contains a table that presents the rules used for section categorisation and two figures that represent the distribution of section titles in the full OA set, as well as in the 100-article manually-checked set, demonstrating that both sets comply with Zipf's law, and demonstrating that the 100-article test set is representative of the complete set. Table S1. Rules Used for Section Categorisation. Figure S1. Section Title Frequencies in the Open Access set. Figure S2. Section Article Frequencies in the 100 Article Test Set. [file 13326_2015_3_MOESM1_ESM.docx]

Table S1: Rules Used for Section Categorisation

| **Section Category** | **Rules** |
| --- | --- |
| Conclusion & Future Work | (conclusion\|key message\|future\|summary\|recommendation\| implications for clinical practice\|concluding remark) |
| Case Report | (case study report\|case report\|case presentation\|case description\|case summary\|case history\| (\d)+\. case\|^ case (\d)+$\|^case$\|^cases$) |
| Supplementary Data | 1.Check a given title for (supplementary\|supporting information\|supplemental\|web extra material)  2. Check if the content of the <footnote> matches with (supplementary) |
| Keyword | (keyword\|key word\|key term\|index\|ocis code\|mesh\|accession\|search term) |
| Abbreviation | 1.Extract content of the <glossary> element from a given xml document  2.(abbreviation\|glossary) |
| Introduction & Background | (introduction\|background\|related literature\|literature review\| objective\| purpose of this study\|study (purpose\|aim\|aims))\| (\d)+. (purpose\|aims\|aim)\|(aims\|aim\|purpose) of the study) \| (the\|drug\|systematic\|book) review\|review of literature\|related work\| recent advance)\|(^aim$\|^aims$\|^purpose$\|^purposes$\|^purpose/aim$\| ^purpose of study$\|^review$\|^reviews$\|^minireview$) |
| Materials & Methods | (method\|material\|experimental procedure\|implementation\| methodology\|treatment\|statistical analysis\|(\d)+. Experimental\| experimental (section\|evaluation\|design\|approach\|protocol\|setting\|set up\|investigation\|detail\|part\|pespective\|tool)\|study protocol\| construction and content\|experiment (\d)+\|analysis\|utility\|design\| (\d)+\. Theory\|theory and\|theory of)\| (  ^experiments$\|^experimental$\|^the study$\|^(\d)+. the study$\| ^protocol$\|^protocols$\|^theory$) AND NOT (supplement) |
| Results | (result\|finding\|diagnosis) |
| Discussion | (discussion\|management of\|(\d)+. management\|safety and tolerability\|limitations\|perspective\|commentary\|(\d)+. comment\|^management$\|^comment$\|^comments$) |
| Acknowledgement&Funding | 1.Extract content of the <ack> element from a given xml document 2.Check if the content of the <footnote> matched with (financial disclosure\|support\|fund\|grant\|thank)  3.(funding\|acknowledgment\|acknowledgement\|acknowledgement\|acknowlegement\|open access\|financial support\|grant\|author note) |
| Author Contribution | 1.(author\| authors'\| author's) contribution  2.Check if the content of the <footnote> element matched with (fn-type=”con”) |
| Competing Interest | 1. (competing interest\|(conflict\|conflicts) of interest\|disclosure\|declaration)  2. Check if the content of the <footnote> element matched with (fn-type=”conflict”) |
| References | Extract content of the <ref-list> element from a given xml document |
| Appendix | (appendix\|appendices) |
| Tables | Extract content of all <table-wrap> elements from a given xml document |
| Figures | Extract content of all <fig> elements from a given xml document |

\d: denotes digits, + denotes one or more times, | denotes OR, ^ denotes beginning of the string, $ denotes end of the string. Patterns are case insensitive and plural forms of the words are taken into account.

Figure S1: Section Title Frequencies in the Open Access set

Figure S2: Section Article Frequencies in the 100 Article Test Set

We checked whether the OA set and the 100-article manually examined test set both comply with Zipf’s law. Zipf’s law is a commonly used model of the distribution of terms in a corpus of natural language utterances. It states that the frequency of any word is inversely proportional to its rank. If a corpus complies with Zipf’s law, this shows that that the corpus is not biased. Figure 1 and Figure 2 show that distribution of the section title frequencies in both the OA set and the 100-article test set comply with Zipf’s law. Hence neither set is biased. In addition, Figure 2 shows that the test set is a good representation of the whole OA-PMC set. The source data for these graphs are the section titles extracted from the OA set and the 100-article manually examined test set (the source data is provided in SectionTitles.xltx).
